# The TRIM-NHL Protein LIN-41 Controls the Onset of Developmental Plasticity in *Caenorhabditis elegans*

**DOI:** 10.1371/journal.pgen.1004533

**Published:** 2014-08-28

**Authors:** Cristina Tocchini, Jeremy J. Keusch, Sarah B. Miller, Susanne Finger, Heinz Gut, Michael B. Stadler, Rafal Ciosk

**Affiliations:** 1 Friedrich Miescher Institute for Biomedical Research, Basel, Switzerland; 2 University of Basel, Basel, Switzerland; 3 Swiss Institute of Bioinformatics, Basel, Switzerland; University of Cambridge, United Kingdom

## Abstract

The mechanisms controlling cell fate determination and reprogramming are fundamental for development. A profound reprogramming, allowing the production of pluripotent cells in early embryos, takes place during the oocyte-to-embryo transition. To understand how the oocyte reprogramming potential is controlled, we sought *Caenorhabditis elegans* mutants in which embryonic transcription is initiated precociously in germ cells. This screen identified LIN-41, a TRIM-NHL protein and a component of the somatic heterochronic pathway, as a temporal regulator of pluripotency in the germline. We found that LIN-41 is expressed in the cytoplasm of developing oocytes, which, in *lin-41* mutants, acquire pluripotent characteristics of embryonic cells and form teratomas. To understand LIN-41 function in the germline, we conducted structure-function studies. In contrast to other TRIM-NHL proteins, we found that LIN-41 is unlikely to function as an E3 ubiquitin ligase. Similar to other TRIM-NHL proteins, the somatic function of LIN-41 is thought to involve mRNA regulation. Surprisingly, we found that mutations predicted to disrupt the association of LIN-41 with mRNA, which otherwise compromise LIN-41 function in the heterochronic pathway in the soma, have only minor effects in the germline. Similarly, LIN-41-mediated repression of a key somatic mRNA target is dispensable for the germline function. Thus, LIN-41 appears to function in the germline and the soma via different molecular mechanisms. These studies provide the first insight into the mechanism inhibiting the onset of embryonic differentiation in developing oocytes, which is required to ensure a successful transition between generations.

## Author Summary

Reprogramming into a naïve, pluripotent state during the oocyte-to-embryo transition is directed by the oocyte cytoplasm. To understand how this reprogramming is controlled, we searched for *C. elegans* mutants in which the activation of embryonic genome, a landmark event demarcating the switch from a germline- to embryo-specific transcription, is initiated precociously in germ cells. This screen identified a novel function for LIN-41, a member of the TRIM-NHL protein family, in preventing a premature onset of embryonic-like differentiation and teratoma formation in developing oocytes, thus ensuring a successful passage between generations. This is the first example of such a regulator in cells that are poised for embryonic development. Interestingly, the majority of molecular “roadblocks” to reprograming that have been identified so far are epigenetic regulators. However, we propose that, at least in germ cells, LIN-41-like regulators may fulfill an analogous role in the cytoplasm, which has possible implications for the generation of human pluripotent stem cells.

## Introduction

There is a special relationship between germ cells and pluripotency, *i.e*,. the ability to adopt alternative cell fates. First, germ cells transmit the pluripotent potential to recreate all types of cells in a new individual. Second, germ cells give rise to pluripotent cell lines such as embryonic germ or carcinoma cells and oocyte cytoplasm has the capacity to reprogram somatic nuclei [1], [2]. Finally, in disease, germ cells can abnormally differentiate into diverse somatic cell types, forming teratomas. However, during normal development, the ability to differentiate into all three embryonic germ layers is restricted to the cells of the early embryo. Combined, these observations suggest that the reprogramming potential of germ cells is kept at bay by repressive mechanisms. Depletion of several chromatin modifiers, either alone or combined with an ectopic overexpression of somatic cell fate-specifying transcription factors, can induce reprogramming of *C. elegans* germ cells into somatic cells [3]–[5]. The loss of these factors appears to primarily impact proliferating (pre-meiotic) germ cells and affects chromatin-based regulation. In contrast, our previous work in the same animal demonstrated that a conserved RNA-binding protein, GLD-1/Quaking, prevents teratomatous differentiation of post-mitotic germ cells [6], [7]. Importantly, in *gld-1* mutants, the germline-to-soma transition is accompanied by a precocious onset of embryonic (or zygotic) genome activation (EGA), suggesting a causal connection between EGA and pluripotency. In other animals, the connection between EGA and pluripotency has been also postulated based on the temporal correlation between EGA and the acquisition of a pluripotent chromatin landscape [8], [9].

These observations prompted us to examine whether new regulators of pluripotency can be identified based on a precocious onset of EGA in the germline. Here, we report the discovery of one such novel regulator of pluripotency, LIN-41/TRIM71. LIN-41 belongs to the TRIM-NHL protein family [10]. These proteins contain a TRIpartite Motif (TRIM) consisting of a RING finger domain (commonly endowing a protein with E3 ubiquitin ligase activity, for example [11]–[13]), two B-Box motifs and a coiled-coil domain. Additionally, they also carry six so-called NHL repeats (named after *N*CL-1, *H*T2A and *L*IN-41) and may contain a filamin domain, which have been implicated in both protein-protein and protein-RNA interactions [13]–[17]. Consistently, different molecular functions have been attributed to LIN-41-like proteins, but many questions remain open; for example, it is not clear whether all the domains function together and/or are used in a tissue context-dependent manner [11], [14], [18]–[20]. The TRIM-NHL family includes well-known regulators of self-renewal and differentiation. For example, in *Drosophila melanogaster*, Brat inhibits neuroblast self-renewal, cell growth and ribosome synthesis in the larval brain [21]–[24] and Mei-P26 restricts growth and proliferation in the ovarian stem cell lineage [25]. Defects in TRIM-NHL proteins have also been associated with human pathologies, for example TRIM32 has been implicated in the Bardet–Biedl Syndrome and the Limb-Girdle Muscular Dystrophy [12], [26], [27]. Recently, human LIN-41 has been shown to promote reprogramming of differentiated cells into induced pluripotent stem cells (iPSCs) [28]. Here, we demonstrate a role for LIN-41 in controlling pluripotency during development of an animal. In *C. elegans*, LIN-41 is a well-known component of the somatic heterochronic pathway, which temporally controls the transition from larval to adult cell fates [29], [30]. The *lin-41* germline phenotype described here indicates that, by preventing the onset of embryonic events in developing oocytes, LIN-41 also ensures a successful transition between generations. However, based on our analyses on both existing and newly created LIN-41 mutations, LIN-41 appears to function in the germline and the soma via two distinct molecular mechanisms. Our study identifies the first cytoplasmic “molecular roadblock” to reprogramming in developing oocytes and we propose it to be required to delay the onset of embryonic differentiation until after fertilization.

## Results

**Figure 1:**
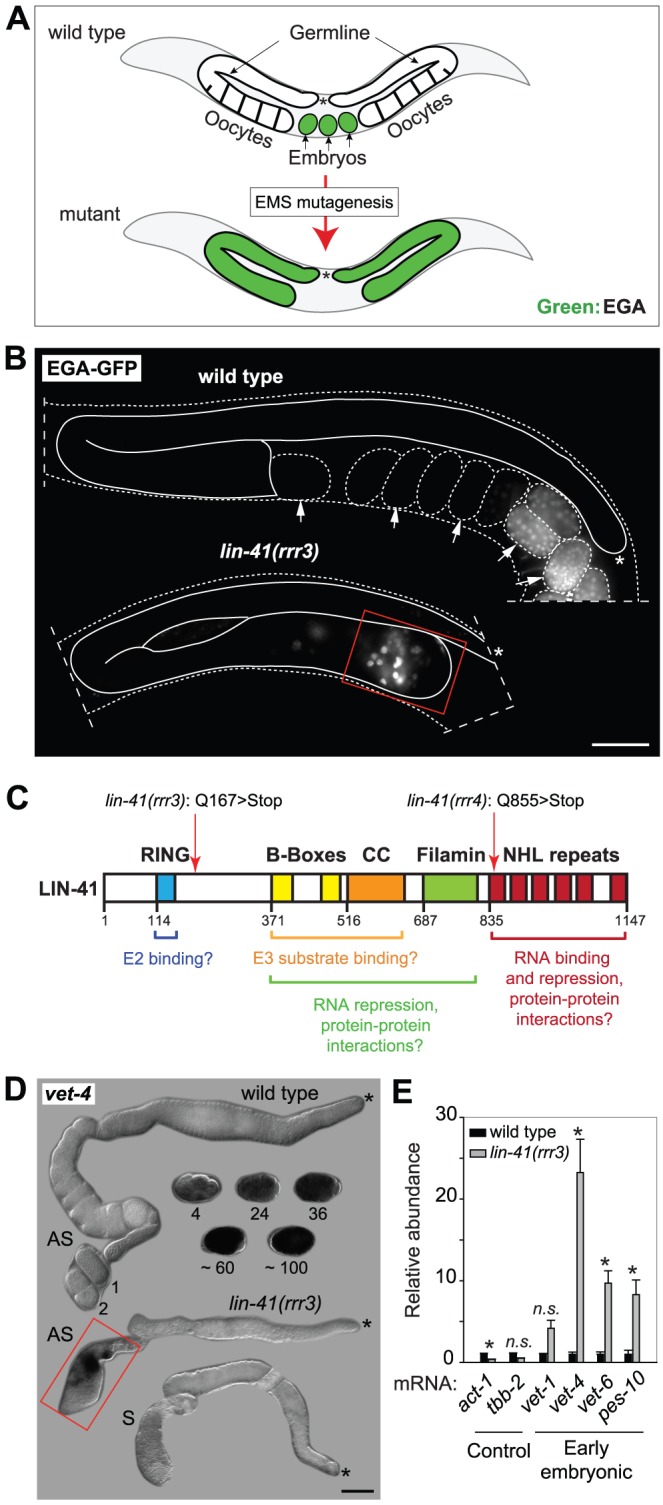
LIN-41 prevents activation of embryonic transcription in the germline. **A.** Summary of a genetic screen to identify mutants inducing EGA in the adult germline. In wild-type, the EGA-GFP reporter (green, marking embryonic transcription) is expressed in embryos. In mutants, this reporter is abnormally expressed in the germline. Asterisks here and in the subsequent figures mark the distal end of the gonad. **B.** Fluorescent micrographs of live animals expressing EGA-GFP. The gonads are outlined with a continuous line, the embryos and animals with dashed lines. Arrows point to selected embryos, the older of which express EGA-GFP. The *lin-41(rrr3)* mutants express EGA-GFP abnormally in the proximal gonad (boxed in red). This phenotype was observed in all examined animals (n>100). Scale bar: 25 µm. **C.** LIN-41 protein domains and their putative functions. Mutations identified in this study are indicated. **D.** *In situ* hybridization against an endogenous EGA transcript, *vet-4*. Shown are light micrographs of gonads and wild-type embryos (at the indicated stages), which were hybridized with antisense (AS) or sense (S) probes for the *vet-4* mRNA. In contrast to the wild-type gonads, *vet-4* mRNA was detected in the proximal region of all *lin-41(rrr3)* gonads (boxed in red; n = 20). Scale bar: 20 µm. **E.** Detection of additional EGA transcripts by RT-qPCR. “Early embryonic”: mRNAs normally expressed in the early embryo following EGA. Each bar represents the mean of three independent biological replicates, the error bars represent the standard error of the mean (SEM) and the significance of the differences has been calculated with the Student's t-test (symbols: “*”, p<0.05; “n.s.”, not significant).

To understand how the onset of pluripotency is controlled during *C. elegans* development, we executed a genetic screen to identify factors that prevent EGA in the adult germline. To monitor EGA, we created a strain expressing GFP from an early embryonic promoter, *vet-4* (very early transcript 4) [31], [32]. Thus, to identify novel regulators of developmental plasticity, we searched for mutants expressing the EGA-GFP in the adult germline (Figure 1A). In addition to a new allele of *gld-1*, this screen yielded two mutants that, in contrast to the embryo-specific EGA-GFP expression in wild-type animals, expressed EGA-GFP within the gonads (Figure 1B). Several lines of evidence suggested that the phenotype of the two mutant strains was caused by alterations in the same gene, *lin-41* (Figures 1C and S1A–D). In these mutants (alleles *rrr3* and *rrr4*, Figure 1C), the EGA-GFP expression was restricted to the proximal region of the oogenic germline (Figure 1B). Consistent with this, RNAi-mediated depletion of *lin-41* resulted in a similar expression of EGA-GFP in the gonad (Figure S1C) and a transgenic construct expressing LIN-41 fully rescued the germline defects of *lin-41(rrr3)* animals (Figure S1D). To further examine the role of LIN-41 in controlling EGA, we verified that the endogenous *vet-4* is also abnormally transcribed in *lin-41(rrr3)* gonads. Indeed, by *in situ* hybridization, we could detect *vet-4* to be expressed in the proximal gonads of *lin-41(rrr3)*, but not wild-type animals (Figure 1D). Next, to examine the extent of embryonic-like transcription in *lin-41(rrr3)* gonads, we monitored the levels of *vet-4* and other additional early embryonic transcripts by reverse transcription and quantitative PCR (RT-qPCR) (Text S1). We found that these transcripts were expressed in mutant, but not wild-type gonads (Figure 1E), further demonstrating that, in *lin-41* mutants, embryonic transcription is prematurely activated in the germline. Importantly, we detected no obvious changes in levels or expression pattern of GLD-1 in *lin-41(rrr3)* gonads (Figure S2), suggesting that the gonadal phenotype of *lin-41* mutants is not caused by defective expression of GLD-1.

**Figure 2:**
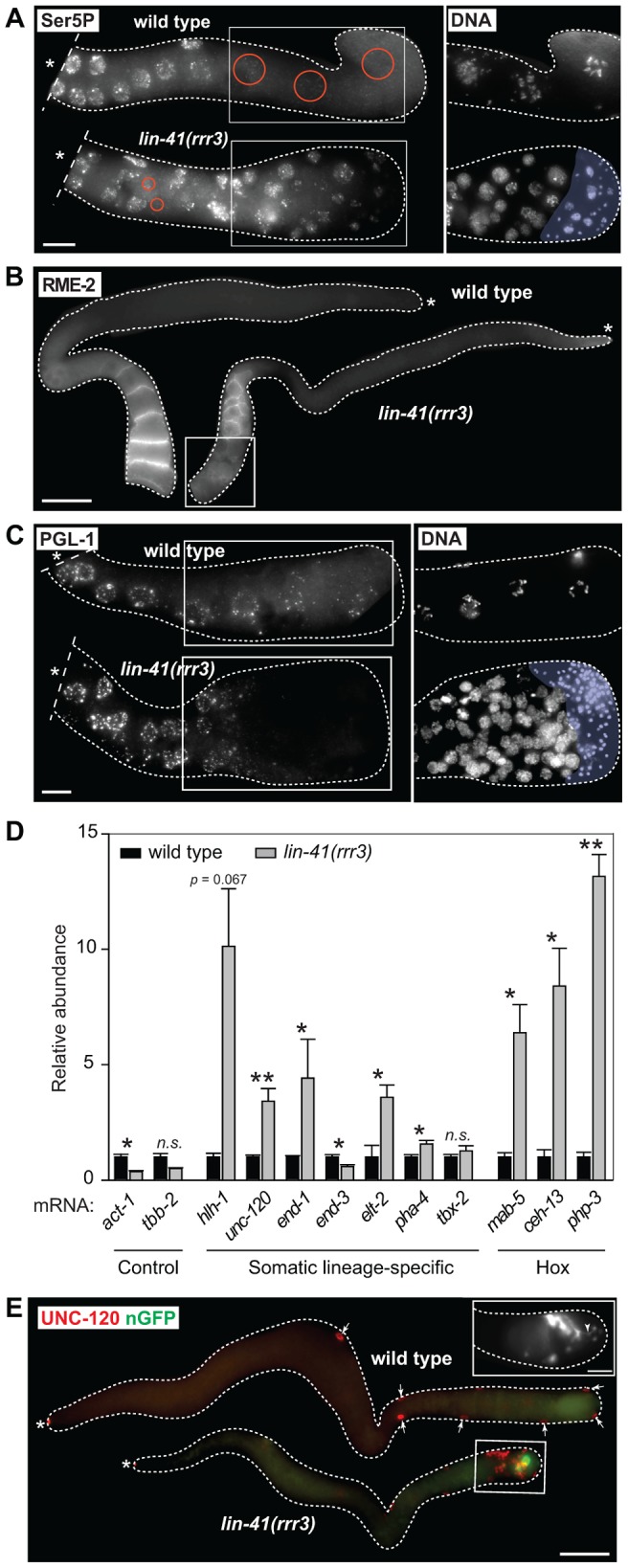
Cells in the proximal regions of *lin-41* gonads lose germline characteristics and differentiate into somatic cells, forming a teratoma. **A.** Fluorescent maximum intensity projections of gonads immunostained for the transcription-activating phosphorylation of Ser5 within the Pol II CTD (Ser5P). This phosphorylation was absent in the most-proximal wild-type gonads, but was present in all corresponding *lin-41(rrr3)* gonads (n>40). Encircled in red are nuclei containing low or no Ser5P. The corresponding DAPI-stained nuclei, from the boxed areas, are on the right. The proximal-most *lin-41* gonad contains sperm (lightly colored in A and C), which explains the lack of Ser5P in this region. Scale bar: 10 µm. **B.** Fluorescent maximum intensity projections of gonads immunostained for an oocyte-expressed protein, the yolk receptor RME-2. In wild-type gonads, RME-2 is expressed in developing oocytes. In *lin-41(rrr3)* gonads, RME-2 is expressed in oocyte-like cells but is absent from the most proximal cells (boxed). Scale bar: 25 µm. **C.** Fluorescent maximum intensity projections of gonads immunostained for a germline-specific protein, PGL-1. PGL-1 (concentrated in RNA/protein granules) is present throughout the wild-type gonad but is eliminated in the proximal *lin-41(rrr3)* gonad (all gonads, n = 25). The corresponding DAPI-stained nuclei from the boxed areas are on the right. Scale bar: 10 µm. **D.** Detection of somatic cell-specific transcripts by RT-qPCR. “Somatic lineage-specific” indicates mRNAs expressed in somatic cell lineages: muscle (*hlh-1*, *unc-120)* or pharynx/gut (*end-1*, *end-3*, *elt-2*, *pha-4* and *tbx-2*). “Hox” genes direct various aspects of somatic development. Each bar represents the mean of three independent biological replicates, the error bars represent the SEM and the significance of the differences has been calculated with the Student's t-test (“*”, p<0.05; “**”, p<0.01; “n.s.”, not significant). **E.** Fluorescent maximum intensity projections of gonads immunostained for the muscle-lineage marker UNC-120 and for a neuronal GFP reporter (nGFP). Arrows point to UNC-120-containing cells of the somatic gonad. In contrast to wild-type, UNC-120 and nGFP-expressing cells (boxed) are present within the *lin-41(rrr3)* germline. The inset shows nGFP-expressing cells from a different *lin-41(rrr3)* gonad, which extend long, neuronal-like processes (arrowhead). Scale bar: 50 µm.

In wild-type animals, Pol II-dependent transcription is repressed in oocytes, which is seemingly at odds with the embryonic-like transcription in the proximal gonads of *lin-41* animals. To investigate this potential discrepancy, we examined the transcription-initiating phosphorylation of serine 5 (Ser5P) within the C-terminal domain (CTD) of Pol II [33]. In contrast to wild-type gonads, Ser5P was detected in the majority of the cells in the proximal gonads of *lin-41(rrr3)* animals (Figure 2A), indicating ongoing Pol II-dependent transcription. Apart from EGA, the onset of embryonic development is marked by the degradation of germline mRNAs and proteins [34]. To examine this aspect of the germline-to-soma transition in *lin-41* animals, we followed the expression levels of RME-2, a yolk receptor present in oocytes [35], and PGL-1, a constitutive component of germ cell-specific RNA/protein granules [36]. In contrast to wild-type animals, which express RME-2 in developing oocytes and PGL-1 throughout the germline, we found that both proteins were absent from the proximal *lin-41(rrr3)* gonads (Figure 2B–C), indicating that cells in this gonadal region lose germline identity. To test this further, we monitored expression of several transcripts that are normally expressed in somatic lineages. By RT-qPCR (Text S1), we found that several of these transcripts (for example the myogenic *hlh-1*/MyoD) were abnormally expressed in *lin-41(rrr3)* gonads (Figure 2D). Additionally, we examined the expression of several *hox* genes, which control the positional identities of cells during animal body formation [37]. While the *hox* transcripts were not expressed in wild-type gonads, they were strongly expressed in *lin-41(rrr3)* gonads (Figure 2D). Finally, we analyzed the expression of the muscle lineage markers UNC-120 and muscle myosin, the intestine lineage marker ELT-2 and a GFP reporter driven from a pan-neuronal *unc-119* promoter (nGFP). We observed that *lin-41(rrr3)* gonads contained numerous cells expressing muscle and neuronal markers (Figures 2E and S3; 44/45 examined gonads contained cells expressing UNC-120, 10/18 cells expressing muscle myosin, and 57/57 cells expressing the nGFP). Only few gonads contained ELT-2-expressing cells (3/35 gonads and only in few cells), which might reflect a competitive advantage of some differentiation programs in the *lin-41* teratoma. During embryogenesis, most body-wall muscles of an adult animal are specified by the transcription factor PAL-1/CDX [38]. The PAL-1-dependent transcription is relatively well understood and involves the activation of its direct targets, such as HLH-1/MyoD and UNC-120/SRF [39]. In wild-type oocytes, expression of PAL-1 is insufficient for the induction of its target genes (Figure S4A–B) [40]. Nevertheless, we observed that the numbers of UNC-120-expressing cells in *lin-41(rrr3)* gonads were significantly reduced upon *pal-1* RNAi (Figure S4B). Thus, the differentiation into muscles in *lin-41* gonads appears, at least partly, to mimic the pathway driving muscle formation in embryos. Together, these findings indicate that *lin-41* germ cells in the proximal gonad undergo a dramatic reprogramming, which results in the acquisition of an embryonic-like state and teratomatous differentiation.

**Figure 3:**
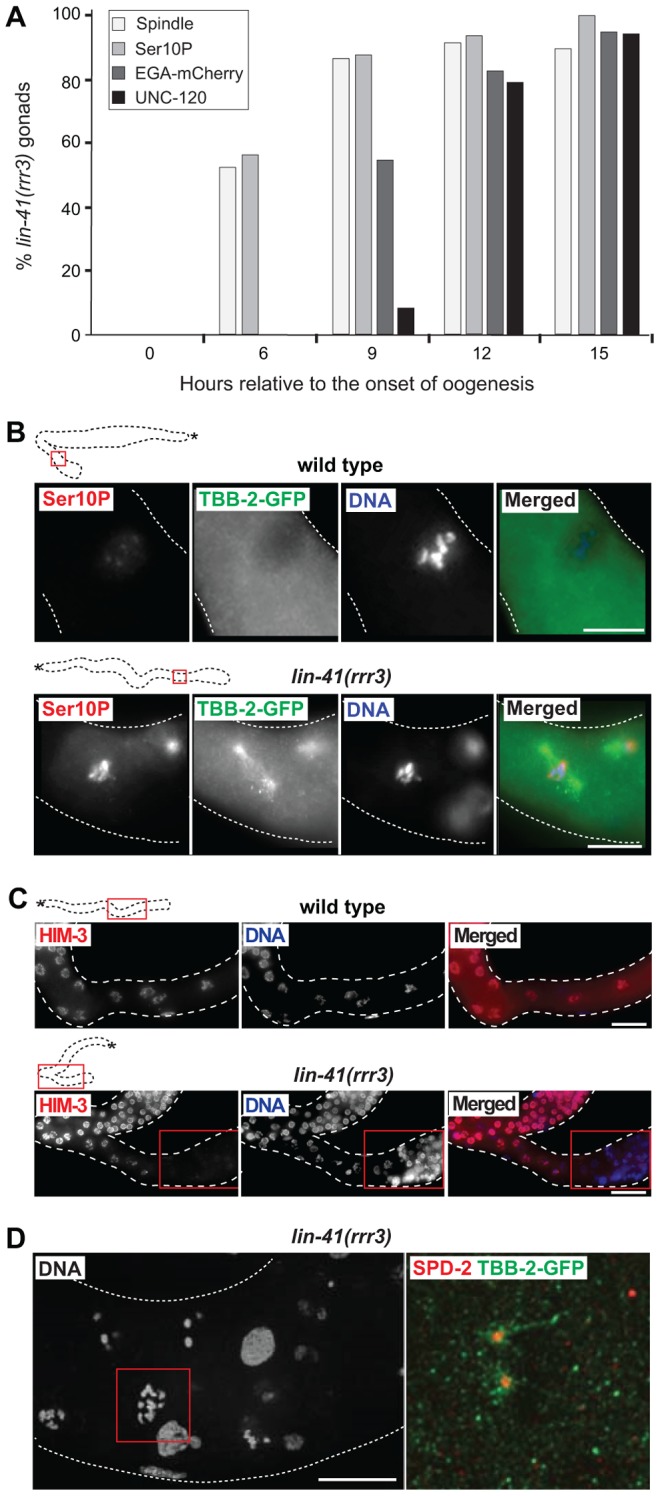
LIN-41 inhibits the transition from meiosis to mitosis in developing oocytes. **A.** Time-course of EGA, mitotic chromosome condensation, spindle formation and somatic-like differentiation in *lin-41(rrr3)* gonads. The numbers indicate the fractions of *lin-41(rrr3)* gonads expressing EGA-mCherry (here the *vet-4* promoter drives mCherry-tagged H2B) or assembling mitotic spindles (visualized with GFP-tagged β-tubulin; TBB-2-GFP), which was observed in live animals at the indicated time points. Additionally, these animals were immunostained for a mitotic marker (Ser10P) or UNC-120. At least 30 gonads were examined per each time-point/marker. **B.** Fluorescent maximum intensity projections of selected regions (boxed in red on the schematic gonads) of wild-type and *lin-41(rrr3)* gonads, immunostained for Ser10P and microtubule spindle (TBB-2-GFP), also stained by DAPI. Scale bars: 10 µm. **C.** Fluorescent maximum intensity projections of selected regions of wild-type and *lin-41(rrr3)* central-proximal gonads, immunostained for the meiotic marker HIM-3 and also stained by DAPI. Scale bars: 25 µm. **D.** Confocal images of maximum intensity projections of selected cells in the proximal *lin-41(rrr3)* gonad stained by DAPI, immunostained for the centrosomal component SPD-2 and for TBB-2-GFP, one day after the L4-to-adult molt. In contrast to wild-type gonads (not shown), cells in the proximal *lin-41* gonads contained duplicated centrosomes (red), facilitating the assembly of microtubule spindles (green). Number of observed *lin-41* cells forming a spindle with duplicated centrosomes: 60/60. Scale bar: 10 µm.

To better understand the germline-to-soma transition in *lin-41* animals, we examined cells in *lin-41(rrr3)* gonads in a time-course experiment (Figure 3A). Until immediately after the end of spermatogenesis, the morphology and numbers of germ cells in *lin-41* and wild-type gonads appeared similar. However, concomitantly with the onset of oogenesis, differences between the *lin-41* and wild-type germlines began to emerge. The proximal region of wild-type gonads contained fully-grown oocytes harboring chromosomes arrested at the diakinesis stage of meiosis I. In stark contrast, the proximal region of *lin-41* gonads contained oocyte-like cells that were about to divide, as evidenced by the presence of highly condensed chromosomes (marked by the phosphorylation of histone H3 on serine 10, Ser10P [41], and microtubule spindles (Figure 3A–B). Consistent with entering a mitotic cell cycle, cells in the proximal *lin-41(rrr3)* gonads did not express HIM-3 (Figure 3C), a synaptonemal complex component [42]. Wild-type oocytes eliminate centrosomes, presumably to ensure the correct ploidy in embryos [31], [43], [44]. In contrast, by monitoring a constitutive centrosome component, SPD-2 [45], we found that centrosomes were present in the proximal *lin-41(rrr3)* gonads (Figure S5). These centrosomes could duplicate (Figures 3D and S5) and were able to nucleate microtubule spindles (Figure 3D). Finally, in addition to the cell cycle markers, we monitored expression of an EGA reporter (EGA-mCherry) and the muscle-lineage marker UNC-120 and observed that their expression followed the onset of mitosis (Figure 3A). Taken together, the absence of LIN-41 leads to the elimination of germline proteins, induction of EGA, a change from the meiotic to the mitotic cell cycle and somatic-like differentiation. Thus, rather than completing oogenesis, cells in the proximal *lin-41* gonads execute events that, in wild-type development, only occur during embryogenesis.

**Figure 4:**
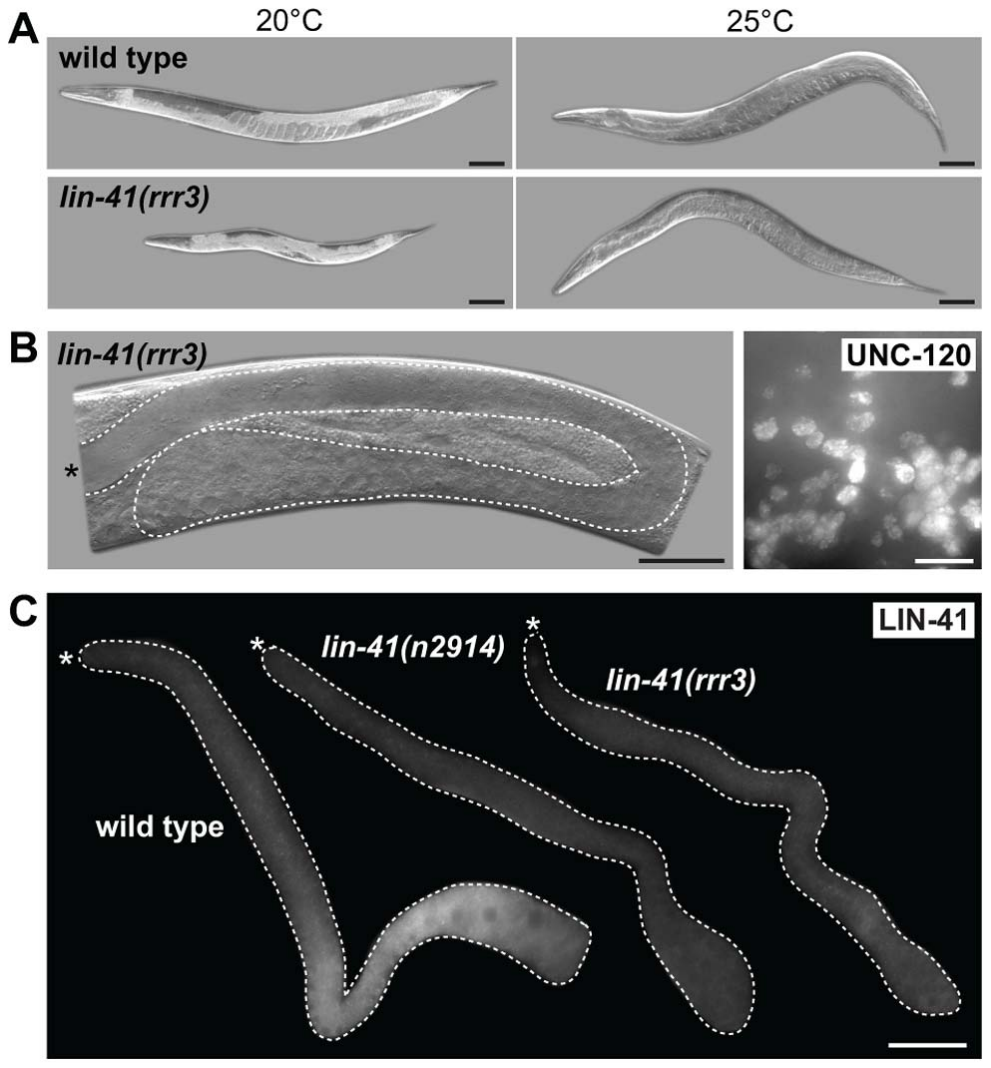
LIN-41 controls the germline-to-soma transition autonomously in the germline. **A.** DIC micrographs of live animals, either wild-type (upper panels) or *lin-41(rrr3)* (lower panels), carrying a GFP-LIN-41 rescuing transgene driven by a heat-shock promoter (*hsp-16.41*). Control animals grown at 20°C are shown on the left and animals grown at 25°C (allowing leaky expression form the heat-shock promoter) on the right. Scale bars: 50 µm. **B.** Left panel: a DIC micrograph of a live *lin-41(rrr3)* animal grown at 25°C. The gonad is outlined with a dashed line. Note the absence of oocytes and the presence of a proximal germline tumor. Scale bar: 50 µm. Right panel: fluorescent maximum intensity projections of a small area from a proximal gonad immunostained for the muscle-lineage marker UNC-120, indicating teratoma formation in *lin-41(rrr3)* animals grown at 25°C. Scale bar: 10 µm. **C.** Gonads of the indicated genotypes immunostained for LIN-41. Scale bar: 50 µm.

In addition to the germline defects, *lin-41(rrr3)* animals displayed somatic abnormalities: decreased size (dumpy phenotype), appeared sick and occasionally bursted through the vulva. These phenotypes have been extensively described by Slack and colleagues and are caused, at least in part, by a precocious translation of the transcription factor LIN-29 [29]. To determine whether the gonadal phenotype reflects LIN-41 function in the germline, or it is indirectly caused by the loss of LIN-41 in the soma, we created a transgene driving *lin-41* expression from a heat-shock promoter (*hsp-16.41*). Due to a general insensitivity of germ cells to the heat-shock promoter-driven expression [46], this transgene was not expressed in the germline but, when crossed into the *lin-41(rrr3)* mutant background and cultivated at an elevated temperature (24–25°C, which is apparently enough to drive sufficient expression of *lin-41* in the soma), it rescued the somatic *lin-41* defects (the transgenic animals no longer appeared sick or short; Figure 4A). Despite the somatic rescue, these animals still developed teratomas (Figure 4B, 50/50 examined animals), suggesting that, in controlling the germline-to-soma transition, LIN-41 functions autonomously in the germline. To examine this further, we immunostained gonads using antibodies raised against LIN-41 and found that, indeed, LIN-41 was present in the cytoplasm of germ cells starting from the late pachytene stage and culminating in the fully-grown oocytes (Figure 4C). Interestingly, LIN-41 was often absent from the most-proximal oocytes (Figures S1D and S6), suggesting a possible connection between oocyte maturation and/or ovulation and LIN-41 levels. LIN-41 expression was limited to the oogenic germline (*i.e.*, it was absent in sperm, *e.g.*, S1D), suggesting that the germline-to-soma transition in *lin-41* gonads is caused by the loss of LIN-41 function in the developing oocytes.

In the soma, LIN-41 is thought to associate with and repress the mRNA encoding a transcription factor, LIN-29, and LIN-29 depletion suppresses the somatic defects of *lin-41* mutants [29]. In contrast to these observations in the soma, *lin-29* mRNA appears to be either poorly or not at all expressed in the germline (our unpublished results and [47]). Consistently, we found that RNAi-mediated depletion of LIN-29 did not suppress the germline defects of *lin-41(rrr3)* mutants (though, it suppressed the somatic defects, as expected [29]). We obtained similar results in *lin-41; lin-29* double mutants (Figure S7). Thus, LIN-41 may function in the germline and soma via distinct targets and/or mechanisms.

**Figure 5:**
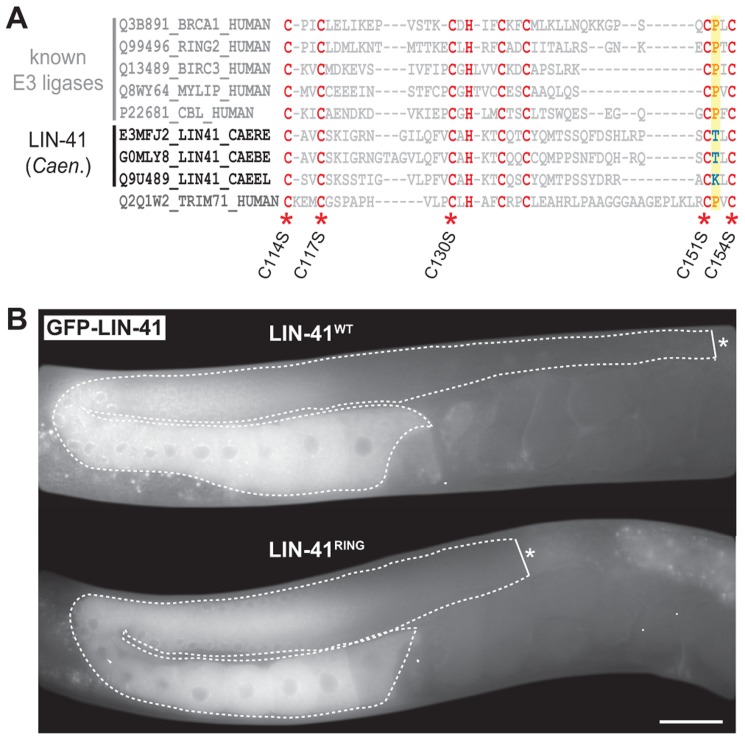
LIN-41 does not function as an E3 ubiquitin ligase. **A.** Sequence alignment of the mammalian TRIM71/LIN-41 RING domain with those from *Caenorhabditis* species and different known E3 ubiquitin ligases. Asterisks indicate the position of conserved cysteines (*C. elegans*: C114, C117, C130, C151, C154), which have been mutated to serines (S) to disrupt the domain (LIN-41^RING^). A highly conserved proline residue (P, highlighted in yellow), which is critical for canonical E3-E2 interactions, is absent in the nematode LIN-41 proteins. **B.** Fluorescent micrographs of live animals expressing wild-type (LIN-41^WT^) and mutated (LIN-41^RING^) GFP-tagged LIN-41 in the *lin-41(rrr3)* mutant background. The gonads are outlined with a dashed line. No differences in the distribution or in the levels of the two proteins have been observed (n>50). Scale bar: 20 µm.

The domain structure of LIN-41 reflects the diversity of functions that have been associated with TRIM-NHL proteins (Figure 1C). Several of these proteins function as E3 ubiquitin ligases, which require a functional RING domain [10], [30]. However, a sequence alignment of the *C. elegans* LIN-41 RING domain with those of other *Caenorhabditis* species indicates that a highly conserved proline, critical for canonical E3-E2 interactions [48], is not found in the nematode LIN-41 RING domains (Figure 5A). Moreover, mutating five cysteine residues that are critical for the RING domain zinc finger structure (C114S, C117S, C130S, C151S, C154S; Figure 5A) resulted in a protein that rescued both somatic and germline defects of *lin-41(rrr3)* animals (Figure 5B). Although we cannot rule out the possibility that LIN-41 associates with additional factors to regulate ubiquitination, these results suggest that the nematode LIN-41 does not function as a direct E3 ubiquitin ligase.

**Figure 6:**
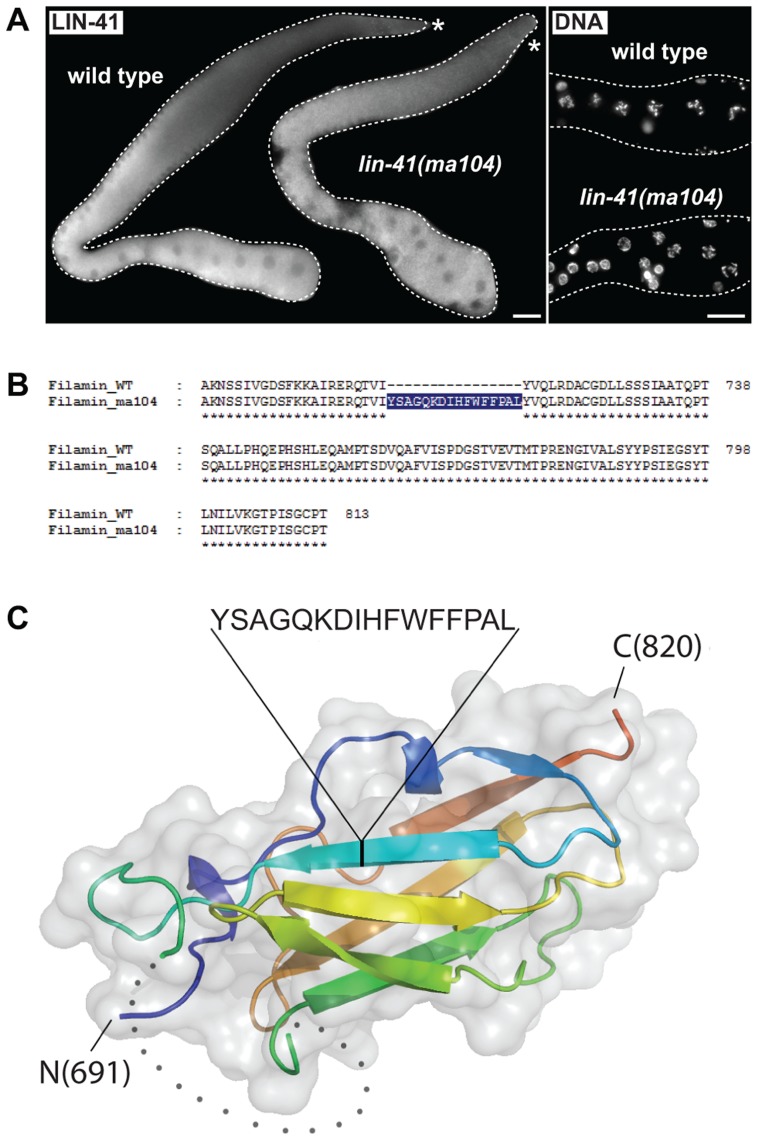
The filamin domain of LIN-41 is not essential for the germline function. **A.** Left: fluorescent micrographs of gonads of the indicated genotypes immunostained for LIN-41. Right: corresponding maximum intensity projections of DAPI staining of the proximal-most region of the gonads shown on the left. Scale bars: 25 µm. **B.** Amino acid sequences of the filamin domain for wild-type and *lin-41(ma104)* mutant (derived from a cDNA sequence). **C.** Crystal structure of the LIN-41 filamin domain. The domain is presented as a cartoon model in rainbow colors from blue (N-terminus) to red (C-terminus) with a transparent surface. The position and sequence of the 16-residue insert present in the *lin-41(ma104)* allele in the second β-strand is highlighted. The disordered sequence stretch 730–757 which is not included in the model is displayed as grey dots.

The coiled-coiled, filamin and NHL domains of the human homolog of LIN-41, TRIM71, constitute the minimal region responsible for binding mRNA and inhibiting translation [19], which is the function attributed to the *C. elegans* LIN-41 in the soma [29]. One previously isolated *lin-41* allele, *ma104*, is a transposon insertion into the sequence coding for the filamin domain [29]. Intriguingly, *lin-41(ma104)* mutants display the somatic defects, but the animals are apparently fertile [29]. We confirmed this and, although the brood size in *lin-41(ma104)* animals is decreased [29], we found no obvious differences in the levels or localization of the germline LIN-41^ma104^ (Figure 6A) and no evidence for a precocious EGA in the gonads (0/35 worms were expressing the EGA reporter in their gonads). To understand the effect of the *ma104* mutation on the LIN-41 protein, we examined the cDNA product of the *lin-41(ma104)* allele. We found that the *ma104* mutation resulted in an insertion of 16 amino acids into the filamin domain (Figure 6B). To gain a mechanistic insight into the *ma104* mutation, the filamin domain (residues 691–821) was subcloned, overexpressed in bacteria, and the protein purified to homogeneity. The protein was crystallized and the structure determined at high resolution (1.68 Å; for data collection and refinement statistics, see Table S1). The final crystallographic model encompasses residues 691–729 and 758–820, whereas a long insert (730–757) that is only found in *Caenorhabditis* LIN-41 protein sequences could not be built due to high flexibility. We found that the LIN-41 filamin structure exhibits a classical immunoglobulin (IG)-like domain fold consisting of seven β-strands arranged in two antiparallel β-sheets (Figure 6C) [49]. A structural search with the LIN-41 filamin domain against the Protein Data Bank (PDB), using DALI, identified the filamin domains most structurally similar to that of LIN-41, which yielded the filamin domains from the *Dictyostelium discoideum* gelation factor, the human TRIM45 and Filamin-A (PDB IDs 1QFH, 1WLH, 2DS4 and 3RGH, respectively) with root-mean-square deviation values for Cα positions between 1.6 and 2.0 Å [50]. Both crystal packing analysis using PISA [51] and SEC MALS experiments of the protein in solution reveal the oligomeric state of this protein domain as monomeric. Importantly, the 16-residue insertion present in the *ma104* allele maps to the mid-section of the second β-strand and is very likely perturbing the filamin IG-like fold (Figure 6B–C). Specifically, the 16-residue insert will prevent completion of one of the β-sheets resulting in solvent access to the hydrophobic protein core of the IG-like β-sandwich and thereby severely destabilizing the fold. This makes it very unlikely that the filamin domain of the *ma104* allele is properly folded to exert its biological function.

**Figure 7:**
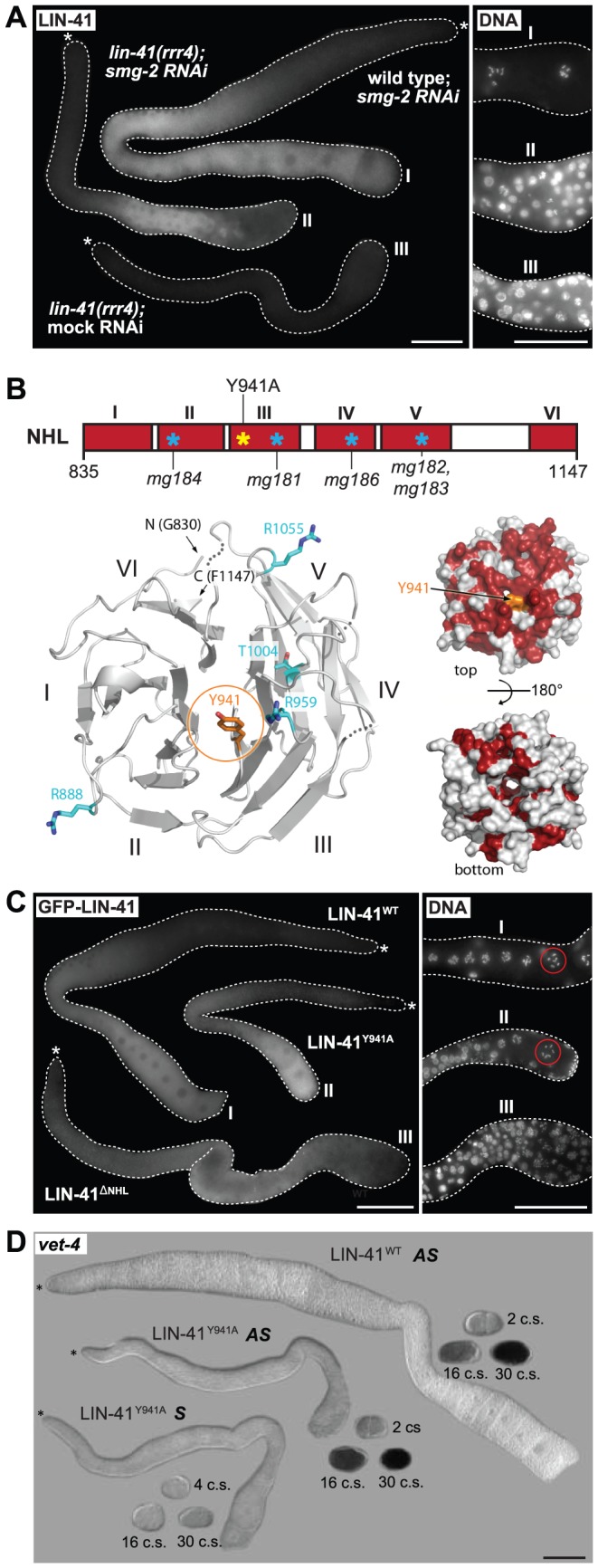
LIN-41 may control the germline-to-soma transition independently from its role in mRNA regulation. **A.** Fluorescence micrographs of *lin-41(rrr4)* gonads stained for LIN-41. Left: suppressing nonsense-mediated mRNA decay (by *smg-2 RNAi*) restores the expression of LIN-41^rrr4^. Right: by maximum intensity projections of DAPI staining, LIN-41^rrr4^ does not rescue the oocyte defects observed in LIN-41-depleted gonads. In contrast to the wild-type gonad containing oocytes in the proximal-most region (I), the gonad expressing LIN-41^rrr4^ (II) (n = 15) accumulates smaller nuclei, which are similar to those in the LIN-41-depleted gonad (III). Scale bars: 50 µm. **B.** Top: a schematic view of the LIN-41 NHL domain and its six β-propellers marked in red (I–VI). Previously identified mutations [29] and our point mutant “LIN-41^Y941A^” are indicated. Bottom left: a homology model of the LIN-41 NHL domain viewed from the electropositive side. The NHL propeller blades are numbered (I–VI) and N-/C-termini are indicated. The residue Y941 is shown in orange atom colors. Known substitution mutations [29] are displayed as sticks in cyan (atom colors). Loops that could not be modeled due to lack of homology are shown as dotted lines. Bottom right: surface representation of the LIN-41 NHL domain homology model in two orientations (rotated by 180° along a horizontal axis). Fully conserved surface-exposed residues are marked in red (see alignment in Figure S8). Y941 (in orange) is in the center of the highly conserved surface patch on the electropositive side of the NHL domain. **C.** Left: gonads expressing the indicated GFP-tagged LIN-41 variants in otherwise *lin-41(rrr3)* gonads. Right: by maximum intensity projections of DAPI staining, LIN-41^ΔNHL^ did not rescue the oocyte defects, as evident by the accumulation of smaller nuclei similar to those in the LIN-41-depleted gonad. In contrast, gonads expressing LIN-41^Y941A^ contained overall normal oocytes and LIN-41^Y941A^ rescued the sterility of *lin-41(rrr3)* animals. At least 50 gonads per strain were examined. Scale bars: 50 µm. **D.** *In situ* hybridization against an endogenous EGA transcript, *vet-4*. Shown are light micrographs of gonads and embryos (at the indicated cell stages, “c.s.”), which were hybridized with antisense (AS) or sense (S) probes for the *vet-4* mRNA. Similarly to the gonads expressing the rescuing LIN-41^WT^, *vet-4* mRNA was absent from the gonads expressing LIN-41^Y941A^. Scale bar: 50 µm.

In the fly Brat and the mammalian TRIM71/LIN-41, the NHL domain is essential for mRNA regulation [14], [19], [52]. One of the *lin-41* alleles reported here, *rrr4*, introduces a premature stop codon within the first NHL repeat (Figure 1C), potentially triggering mRNA degradation via nonsense-mediated mRNA decay (NMD). Indeed, inhibiting NMD (by depleting an NMD component, SMG-2 [53]), restored the wild-type expression pattern and levels of LIN-41^rrr4^ (Figure 7A). LIN-41^rrr4^ is expected to lack the NHL domain and we found that the gonads expressing this LIN-41 variant displayed *lin-41*-like germline and somatic defects (Figure 7A). Thus, the NHL domain appears to be essential for LIN-41 functions in both germ and somatic cells.

The NHL domain structure of Brat forms a six-bladed β-propeller [52]. Several point mutations in Brat and TRIM71 that disrupt mRNA regulation affect residues on the electropositive side of the NHL domain [19], [23], [52], [54] (Figures S8, S9), highlighting the importance of this surface for mRNA regulation. Point mutations in the NHL domain have been also reported in LIN-41 (Figures 7B and S9A–B) [29]. Importantly, although these mutations display defects in the soma, the animals are fertile, suggesting that the mutant proteins fulfill the gonadal functions [29]. To better interpret these mutations, we initially attempted, unsuccessfully, to express the LIN-41 filamin-NHL or a NHL-only domain constructs for protein structure determination. Thus, we created a homology model of the LIN-41 NHL domain based on the crystal structure of the Brat NHL domain. Interestingly, we found that most of the existing point mutations in the LIN-41 NHL domain also affect amino acids residing on the electropositive surface of the NHL domain (Figure 7B). These observations suggest that i) the electropositive surface of the NHL domain plays a conserved function in mRNA regulation and ii) the germline and somatic functions of the NHL domain involve different mechanisms. To explore this further, we introduced an additional mutation (Y941A) on the electropositive surface of the NHL domain (Figures 7B and S9). Potentially, this mutation is more informative than the other existing NHL mutations because mutation of the corresponding residue (Y702A) in TRIM71 is known to abolish mRNA regulation [19]. In contrast to the deletion of the whole NHL domain from otherwise rescuing (FLAG- and GFP-tagged) LIN-41 protein, which, as expected, caused defects in both the soma and the germline, we found that the LIN-41^Y941A^ variant largely suppressed the germline defects and sterility of *lin-41* animals (Figure 7C), including the precocious expression of the endogenous *vet-4* transcript (Figure 7D), though it continued to display the somatic defects. Thus, if the same domains/residues determine mRNA regulation in LIN-41 as in TRIM71, the germline function of LIN-41 might be independent from mRNA binding.

## Discussion

### Cytoplasmic regulators of pluripotency

In contrast to the much-publicized regulation of pluripotency via DNA and chromatin modifications, the potential for cytoplasmic regulation has largely been neglected. Our findings suggest that proteins like LIN-41 and GLD-1 can function in the cytoplasm as molecular “roadblocks” to reprogramming, analogous to the nuclear factors. Similarly, components of P-granules (germline-expressed RNPs) have been recently reported to facilitate maintenance of germline identity in proliferating germ cells, *i.e.*, at the stage prior to GLD-1 expression [55]. Whether teratomatous differentiation in the absence of P-granules reflects precocious activation of embryonic transcription, or has a different etiology, remains to be determined. However, in contrast to P-granules, that impact multiple aspects of RNA metabolism, GLD-1 and LIN-41 are expected to have more specific functions. Intriguingly, the germline-to-soma transition in the absence of LIN-41 or GLD-1 involves similar events: loss of germline proteins, retention of centrosomes, execution of mitosis and activation of the embryonic genome. Two GLD-1 mRNA targets, important for the germline-to-soma transition, encode the CDK-2 partner protein CYE-1/cyclin E and the transcription factor PAL-1/Cdx [6], [31]. However, cyclin E and PAL-1 are co-expressed with LIN-41 in the developing wild-type oocytes [56], suggesting that their expression is not regulated by LIN-41. Thus, GLD-1 and LIN-41 may regulate pluripotency via different targets and/or mechanisms. While GLD-1 directly binds and regulates the expression of its mRNA targets, the molecular function of LIN-41 remains elusive. Our analysis suggests that the germline function of LIN-41 may be independent from mRNA binding, though it does not exclude its role in posttranscriptional regulation, for example as a component of a regulatory RNP. In addition to binding RNA, several TRIM-NHL proteins have been shown to modulate functions of other proteins, for example, by their sequestration (*e.g.*, TRIM3 appears to regulate p21 [13], [17]) or by linking structural proteins (*e.g.*, Wech bridges Talin and ILK for proper embryonic muscle attachment [16]). These interactions depend, at least in part, on the NHL domains of both proteins. Thus, LIN-41 could regulate the germline-to-soma transition by associating, via its NHL domain, with another protein.

### Temporal regulation of the germline-to-soma transition by LIN-41

LIN-41-mediated regulation of cell fate transition between generations is somewhat reminiscent of LIN-41 function in the hetrochronic pathway in the soma. However, specific mutations within the NHL and filamin domains of LIN-41 result mainly in somatic but not germline defects (this study and [29]). In addition, LIN-41-dependent repression of LIN-29 appears to be restricted to the soma. Thus, although LIN-41 regulates developmental transitions in both germ- and somatic cells, it may do so through different molecular mechanisms and/or targets.

In the soma, down-regulation of LIN-41, which is mediated by the *let-7* miRNA, allows terminal differentiation [57], [58]. In the germline, LIN-41 levels decrease in the most-proximal oocytes, so that LIN-41 is absent from the early embryos. An interesting possibility is that the absence of LIN-41 is a trigger for the onset of embryonic differentiation. In order to test this hypothesis, we attempted to over-express LIN-41 from the heat shock promoter in very early embryos. However, LIN-41 was efficiently expressed only after gastrulation (our unpublished observation), *i.e.*, several cell divisions after EGA, making the experiment inconclusive. The down-regulation of LIN-41 occurs while Pol II-dependent transcription is globally repressed in the oocytes, suggesting that the regulation occurs at the mRNA or the protein level. To test for possible regulation at the mRNA level, we expressed a rescuing LIN-41 under the control of a truncated 3′UTR missing most of the sequence, including the *let-7* binding sites [58]. While the expression of this LIN-41 protein started earlier (more distally) in the germline, suggesting posttranscriptional regulation of *lin-41* mRNA in this part of the gonad, LIN-41 was still down-regulated in the oocytes and embryos (our unpublished observation), hinting at a possible regulation at the protein level. If so, testing the functional significance of LIN-41 degradation will require the dissection of regulatory motifs in the protein (for example phosphorylation).

### Mammalian LIN-41/TRIM71 proteins and pluripotency

TRIM-NHL proteins are known to control the proliferation versus differentiation decision in germ- and neuronal stem cell lineages [23], [25], [59] and, intriguingly, *C. elegans* LIN-41 has been recently reported to control the regenerative ability of neurons [60]. While these examples highlight the importance of TRIM-NHL proteins for maintaining homeostasis in self-renewing tissues, these proteins have not previously been implicated in controlling pluripotency during development. In our study, we describe LIN-41 as a critical component of the timing mechanism controlling the oocyte reprogramming capacity. To our knowledge, this is the first example of such a regulator in cells that are ready for embryonic development, providing the initial glimpse into a pathway controlling one of the most fundamental developmental transitions. While the *in vivo* roles of the mammalian LIN-41/TRIM71 are poorly understood, the murine TRIM71 is expressed and functions in developing embryos [61], [62]. TRIM71 is also preferentially expressed in embryonic stem (ES) cells [18], which are derived from pluripotent embryonic cells. In ES cells, TRIM71 represses the expression of Cdkn1, an inhibitor of the cell cycle progression, thereby promoting proliferation [18]. While this role appears opposite to LIN-41 function in the *C. elegans* germline, TRIM71 presumably associates with many mRNAs, making additional roles likely. Intriguingly, the human LIN-41 has been recently shown to facilitate reprogramming of fibroblasts into iPSCs [28]. In this context, LIN-41, combined with several “pluripotency” transcription factors, can circumvent the requirement for c-Myc in reprogramming [28]. c-Myc facilitates reprogramming in several ways, including by inhibiting differentiation [63], and LIN-41 appears to play a similar role by repressing mRNAs encoding pro-differentiation factors [28]. Although the targets and, perhaps, the mechanisms may differ, it is striking that the *C. elegans* LIN-41 appears to fulfill an analogous function in the germline. Thus, dissecting LIN-41 targets and the mechanism are exciting objectives for the future research.

## Materials and Methods

### Nematode culture, mutants, RNAi and transgenic lines

N2 animals were maintained as previously described [64] and were grown at 20°C unless stated otherwise. For alleles and transgenic lines, see Supplemental Material. For RNAi, L1 larvae (L4 for *fog-2*), grown at 25°C, were fed with bacteria expressing dsRNAs (targeting *lin-41*, *pal-1*, *fog-2* or *smg-2* from the Open Biosystem library or *lin-29* from the Ahringer library) and screened one day after the L4-to-adult molt in the same (*lin-41*, *pal-1* and *lin-29*) or in the second generation (*fog-2* and *smg-2*). A bacterial strain carrying an “empty” vector was used as a negative control (mock RNAi).

### Mutagenesis and whole genome sequencing

EMS mutagenesis [64] was performed on a strain (# 1284, see Text S1) carrying the EGA-GFP (integrated at two chromosomal locations to increase GFP fluorescence). F2 animals derived from ≈10.000 F1s were screened. Candidate mutations were identified as previously described [65]. Each mutant was back-crossed four times against the parental strain before genome sequencing. Genomic DNAs (gDNAs) were isolated using Gentra Puregene Tissue Kit 4 g (Qiagen). DNA libraries were created from 50 ng of gDNA (Nextera DNA kit from Illumina). The sequencing data were generated using Hi Seq 2000 (Illumina).

### Processing of sequence data and detection of sequence variants

Sequence reads were aligned to the May 2008 *C. elegans* assembly (obtained from http://hgdownload.soe.ucsc.edu/goldenPath/ce6/chromosomes/) using “bwa” [66]; version 0.6.1-r104) with default parameters, but only retaining single-hit alignments (“bwa samse -n 1” and selecting alignments with “X0:i:1”). The resulting alignments were converted to BAM format, sorted and indexed using “samtools” [67]; version 0.1.18). In order to quantify contamination by *Escherichia coli*, reads were similarly aligned to a collection of *E. coli* genomes (NCBI accession numbers NC_008253, NC_008563, NC_010468, NC_004431, NC_009801, NC_009800, NC_002655, NC_002695, NC_010498, NC_007946, NC_010473, NC_000913 and AC_000091), which typically resulted in less than 1% aligned reads. Sequence variants were identified using GATK [68]; version 1.5.31) indel realignment and base quality score recalibration, followed by SNP and INDEL discovery and genotyping for each individual strain using standard hard filtering parameters, resulting in a total of six to eight thousand sequence variations in each strain compared to the reference genome. Finally, the number of high quality (score > = 500) single nucleotide substitutions of EMS-type (G/C→A/T transitions [69], not found in other any other mutant strain or in the parent strain (typically less than 1% of the total number of variants per strain) were counted in sequential windows of 1 Mb to identify regions of increased variant density.

### Real-time quantitative PCR on dissected gonads

RNA was isolated from gonads dissected from one day-old (after the L4-to-adult molt) animals. cDNA was synthesized with oligo(dT) primers using the ImProm II Reverse transcription system from Promega according to manufacturer's instructions. cDNA was used for qPCR with the Absolute QPCR SYBR green ROX mix (AbGene) on an ABI PRISM 7700 system (Applied Biosystems). qPCR reactions were performed as previously described [31]. At least one primer in each pair is specific for an exon-exon junction. Human carrier RNA was added to each sample before RNA extraction, allowing normalization to hGAPDH. Standard curves for quantification were generated from a serial dilution of input cDNA for each primer pair. The amount of target present in each replicate was derived from a standard curve; an average was calculated for the triplicates. To compare total mRNA levels, the qPCR results were normalized to human GAPDH and to the wild-type values for each primer pair and fold enrichments were calculated. For primers used, see Text S1.

### Immunostaining, antibodies, RNA *in situ* hybridization and microscopy

Immunostaining experiments were performed as previously described [70] with the following antibodies: PGL-1 [36] (dilution 1∶1000); SPD-2 [45] (“969LA”, 1∶800); GFP (Roche, 1∶700); phospo-Histone H3 Ser10 (“Ser10P”, Millipore, 1∶200); muscle myosin [71] (“5–6”, 1∶2.500); and UNC-120 (courtesy of Michael Krause, 1∶500). Immunostainings against RME-2 [35] (“INT”, dilution 1∶100), GLD-1 [72] (dilution 1∶5) and LIN-41 (courtesy of Helge Grosshans, “4796”, 1∶2.000) were performed as previously described [73] and against the Ser5P of Pol II CTD [74] (“3E8”, 1∶5) according to Seydoux and Dunn [33]. Immunostainings against HIM-3 [42] (courtesy of Monique Zetka, dilution 1∶500) were performed as previously described [75]. Secondary antibodies used in this study: goat anti-mouse IgG alexa-488 (Molecular Probes, 1∶600,), goat anti-rabbit IgG alexa-568 (Invitrogen, 1∶750) and goat anti-rat IgG alexa-568 (Molecular Probes, 1∶500). *In situ* hybridizations against the *vet-4* mRNA were performed as previously described [31]. Unless indicated otherwise, the gonads were dissected from 1 day-old adults. Zeiss AxioImager Z1 microscope equipped with an Axiocam MRm REV 2 CCD camera was used for capturing pictures. Images were then exported into Adobe Photoshop CS4 and processed in an identical manner. A spinning disk multipoint confocal microscope equipped with an EM-CCD Cascade II camera (Photometrics) was used for capturing images for Figure 3D. Pictures were, then, deconvolved with the Huygens software and then processed in Imaris XP 7.1.1.

### LIN-41 antibody

The affinity-purified (ELISA) rabbit anti-LIN-41 antibody (“4796”) was provided by Helge Grosshans (Magdalene Rausch & Helge Grosshans, unpublished data) and created against the VKNLKLSVLISQAESLQSKQIDLQQAIQTATKLMDSSDCDEMVLRQVFEKLASCQMGNEGTEPNNNILNVLMLACQVNEDDRLKFTAPQDGILLNKARQF sequence (residues 587–686). The rabbit was raised by SDIX in Newark, DE, USA.

### LIN-41 variants

The LIN-41 point mutant transgene constructs “RING” and “Y941A” were created from the wild-type LIN-41 transgenic template by site-directed mutagenesis (Stratagene QuikChange method), whereas the deletion construct “ΔNHL” was created via two-step PCR. In any case, Phusion High-Fidelity DNA Polymerase (Fermentas) was used. For primers used see Text S1.

### Cloning, expression and purification of LIN-41

The filamin domain (residues 691–821) of *C. elegans* LIN-41 (isoform B of Q9U489) was cloned into pOPINF [76] using In-Fusion (Clontech Laboratories Inc). The resulting expression construct was transformed into BL21 DE3 cells and the protein expressed via auto-induction at 20°C for 20 hours. Cells were harvested, then resuspended in lysis buffer (50 mM Tris, pH 7.5, 500 mM NaCl, 20 mM imidazole, 0.2% Tween-20) and frozen at −80°C. The cell suspension was thawed and freshly supplemented with Complete EDTA-free protease inhibitors (Roche Diagnostics) and 3 U/ml Benzonase (Sigma) before passing through an Avestin EmulsiFlex-C3 cell disruptor. The clarified lysate was incubated with NiNTA affinity resin (Qiagen) in batch mode and the bound protein eluted in 50 mM Tris, pH 7.5, 500 mM NaCl, 125 mM imidazole. The protein was fractionated on a Superdex 75 HiLoad 16/60 (GE Healthcare) gel filtration column in GF buffer (20 mM Tris, pH 7.5, 200 mM NaCl, 2 mM TCEP and 0.02% NaN_3_). The single peak fraction was pooled and digested overnight at 4°C with 3C protease to remove the N-terminal histidine tag. The released protein tag and 3C protease were removed by a second nickel-affinity step and the untagged filamin domain was further purified over a Superdex 75 column in GF buffer and concentrated to 7.5 mg/ml.

### Crystallization, data collection and structure solution

All crystallization experiments were performed at 20°C using the sitting-drop vapour diffusion method via a Phoenix robot (Art Robbins) dispensing 100 nl drops. Removal of the N-terminal histidine tag from the filamin domain was needed to obtain crystals. The untagged filamin domain readily crystallized in many conditions. Crystals grown in 1.1 M sodium malonate, 0.1 M HEPES, pH 7.0, 0.5% v/v Jeffamine ED-2001, were harvested and cryoprotected in mother liquor containing 25% ethylene glycol. These crystals diffracted to 1.68 Å resolution at the SLS PX-III beamline and belonged to space group C222_1_ with one molecule per asymmetric unit. Diffraction data were integrated and scaled using XDS [77] and the structure was solved by the molecular replacement method using PHASER [78]. Phases from this solution were calculated and used for automatic model building with BUCCANEER [79]. The LIN-41 filamin structure was further improved by the crystallographic simulated annealing routine followed by individual B-factor refinement in PHENIX [80] and several rounds of manual rebuilding in COOT [81] and refinement in BUSTER [82]. The final structure was validated using COOT. Structural images for figures were prepared with PyMOL (http://pymol.sourceforge.net/). Atomic coordinates and structure factors for the LIN-41 filamin domain have been deposited in the PDB with entry code 4UMG.

### Homology modeling

Amino acid sequences of the *C. elegans* LIN-41 (Uniprot Q9U489, 830–1147) and *Homo sapiens* TRIM71 (Uniprot Q2Q1W2, 591–868) NHL domains were submitted to the HHPRED server for homology detection and structure prediction [83]. The structure of the *D. melanogaster* Brat NHL domain (PDB 1Q7F) was the top hit in both searches resulting in very high scores for the LIN-41 NHL domain (Score = 241.22, E-value = 1e-33, 28% sequence identity) and the TRIM71 NHL domain (score = 22.54, E-value = 4.5e-35, 31% identity). The top alignments were edited for minimal local corrections and sent to the HHPRED MODELLER pipeline for modeling. Loops lacking template information for both the *C. elegans* and the human NHL domain models were removed in the final models. Structural figures were prepared using PyMOL (www.pymol.org).

## Supporting Information

Figure S1 Mutations in the *lin-41* gene cause precocious onset of embryonic transcription in the germline. **A.** Following EMS-induced mutagenesis and outcrossing of two similar mutants (*rrr3* and *rrr4*) against the wild-type parental strain, whole genome sequencing uncovered sequence variants clustering on chromosome I. Numbers indicate chromosomes and “M” mitochondrial DNA. Genes containing EMS-type mutations and present within the chromosomal regions highlighted in yellow are listed in the table below. **B.** A summary of candidate mutations from the chromosomal regions highlighted above. The only gene mutated in both strains, *lin-41*, is highlighted in red. **C.** Fluorescent micrographs of live wild-type animals expressing EGA-GFP subjected to either mock or *lin-41 RNAi*. The gonads are outlined with a continuous line and the embryos and animals with dashed lines. Consistent with the phenotype of *lin-41(rrr3)* and *lin-41(rrr4)* mutants, *lin-41(RNAi)* animals (examined at 42 hours post-L1 stage) expressed EGA-GFP in the proximal gonad (boxed in red). This phenotype was fully penetrant (n = 50). Scale bar: 50 µm. **D.** DIC (upper panel) and fluorescent (lower panel) micrographs of live *lin-41(rrr3)* animals expressing the FLAG- and GFP-tagged LIN-41 rescuing construct. The gonads and the embryos are outlined with white dashed lines, the spermatheca with red dashed lines (note the absence of LIN-41 from sperm) and the animals with continuous lines. Early embryonic stages (“c.s.” = cell stage) are indicated. Scale bar: 50 µm.
(EPS)

Figure S2 GLD-1 expression is not altered in *lin-41* mutant gonads. Fluorescent micrographs of wild-type and *lin-41(rrr3)* gonads immunostained for GLD-1. Scale bar = 50 µm.
(EPS)

Figure S3 Differentiation into muscles in *lin-41* teratomas. Fluorescent maximum intensity projections of proximal gonads immunostained for muscle myosin. In contrast to wild-type gonads, which contain myosin only in the somatic sheath cells that envelop the germline, clusters of myosin-expressing cells (arrows) are present within the proximal region of the *lin-41(rrr3)* gonads. Scale bars: 20 µm.
(EPS)

Figure S4 PAL-1 is expressed in both wild-type and *lin-41* gonads. **A.** Fluorescent maximum intensity projections of gonads expressing GFP-tagged PAL-1, dissected from 0.5 day-old animals subjected to either mock or *lin-41 RNAi*. Scale bar: 50 µm. **B.** Upper panel: a simplified view of PAL-1-dependent transcriptional cascade. Lower panels: left: fluorescent maximum intensity projections of *lin-41(rrr3)* gonads stained for UNC-120. Scale bar = 25 µm. Right: the corresponding quantification. The numbers of UNC-120 expressing cells per gonad were significantly reduced upon *pal-1 RNAi* (number of analyzed gonads = 37) compared to mock RNAi (number of analyzed gonads = 20) (p<0.001). The error bars represent the SEM.
(EPS)

Figure S5 Centrosome duplication in *lin-41* proximal gonads does not represent an aberrant spermatogenesis. Fluorescent maximum intensity projections of selected gonadal cells immunostained for the centrosomal component SPD-2 (red) and stained by DAPI (green). L4 animals had been subjected to *fog-2 RNAi* and their progeny were used for the experiment. Only animals fully sperm-depleted were analyzed. Upper panels: selected cells from wild-type gonads. Lower panels: selected cells from *lin-41(rrr3)* gonads. Left panels: cells from the distal region of the gonad, where mitotic cells reside and normally exhibit centrosome duplication (arrowheads point to examples of duplicated centrosomes in both wild-type and mutant gonads). Right panels: cells from the proximal region of the gonad. Feminized wild-type proximal gonads contain oocytes where centrosomes have been eliminated; feminized *lin-41(rrr3)* proximal gonads contain centrosomes that have not been eliminated and some of them display duplication (arrowheads). Scale bars = 10 µm.
(EPS)

Figure S6 LIN-41 is down-regulated upon ovulation and is absent from early embryos. Fluorescent micrographs of wild-type gonads and early embryos immunostained for LIN-41 (upper panel) and DAPI (lower panel). Shown are maximum intensity projections. The gonads and the embryos are outlined with dashed lines. Scale bar: 50 µm.
(EPS)

Figure S7 The absence of LIN-29 does not interfere with proximal tumor formation of *lin-41* gonads. DIC micrographs of a live *lin-41(rrr3); lin-29(n546)* double mutant worm; the gonad is outlined with white-dashed lines and the proximal tumor is in red. An asterisk marks the distal end of the gonad and an arrowhead the vulval defects. Scale bar: 50 µm.
(EPS)

Figure S8 Clustal Omega multiple sequence alignment of LIN-41 NHL domain orthologous sequences. The alignment was calculated using the EBI ClustalO server (http://www.ebi.ac.uk/Tools/msa/clustalo/). Fully conserved residues (similarity groups enabled) are shaded in black and mapped onto the surface of the *C. elegans* LIN-41 NHL domain homology model in Figure 7B.
(EPS)

Figure S9 Homology models of the NHL domains of LIN-41 and TRIM71. **A.** Superpositions of the NHL domain homology models of *C. elegans* LIN-41 (left, displayed in grey as in Figure 7B) and *Homo sapiens* TRIM71 (right, in dark purple) onto the X-ray structure of *D. melanogaster* Brat NHL domain (PDB 1Q7F, in blue). Brat residues important for binding of the Pumilio Puf domain are shown as green sticks in atom colors [52]. Residues of the human TRIM71 NHL domain involved in mRNA repression are displayed as sticks in magenta [19]. **B**-**C.** HHPRED alignments between *C. elegans* LIN-41 (B) and *H. sapiens* TRIM71 (C) NHL domain sequences with the sequence of the *D. melanogaster* Brat NHL domain of known structure (PDB 1Q7F). Individual alignments were used to calculate the *C. elegans* and *H. sapiens* NHL domain homology models, respectively. Residues highlighted in the structural superpositions in (A) are indicated in the alignments.
(EPS)

Table S1 Data collection and refinement statistics for the LIN-41 filamin domain. Diffraction data collection statistics for a crystal of the LIN-41 filamin domain are presented in the upper part (Data collection), while statistics for the final structural model and its fit against the experimental data are presented below (Refinement).
(PDF)

Text S1 Supplemental materials and methods. Complete lists of alleles and transgenic lines, RT-qPCR primers, primers used to create the mutated transgenes for *lin-41* and the accession numbers for proteins used in alignments in this study are provided.
(PDF)
