# Supplementary material for: The TRIM-NHL Protein LIN-41 Controls the Onset of Developmental Plasticity in Caenorhabditis elegans
Source: PLoS Genet. 2014 Aug 28;10(8):e1004533. doi: 10.1371/journal.pgen.1004533 (PMC4148191; doi:10.1371/journal.pgen.1004533)
Supplement: Table S1 — Data collection and refinement statistics for the LIN-41 filamin domain. Diffraction data collection statistics for a crystal of the LIN-41 filamin domain are presented in the upper part (Data collection), while statistics for the final structural model and its fit against the experimental data are presented below (Refinement). (PDF) [file pgen.1004533.s010.pdf]

Table S1. Data collection and refinement statistics for the LIN-41 filamin domain

Data collection	
Space group	$C222_1$
Cell const. *a, b, c* [Å]	44.78, 52.11, 101.31
Wavelength [Å]	1.000
Resolution range [Å][a]	50.0-1.68 (1.72-1.68)
Unique reflections	13883
Completeness [%][a]	99.9 (100.0)
Multiplicity	7.0
R_{sym} [%][a]	4.5 (87.8)
$I/\sigma(I)$[a]	20.8 (1.9)
CC(1/2) [%][a]	99.9 (69.3)
Refinement	
Resolution range [Å]	50.0-1.68
Reflections (all)	13854
Reflections (test set)	693 (5.0 %)
R_{crys} [%]	21.9
R_{free} [%]	24.4
**RMSDs**	
Bond lengths [Å]	0.01
Bond angles [°]	1.11
**Ramachandran plot [%]**	
Allowed	100
Outliers	0

[a]Values in parentheses refer to the highest resolution shell
