# Supplementary material for: The TRIM-NHL Protein LIN-41 Controls the Onset of Developmental Plasticity in Caenorhabditis elegans
Source: PLoS Genet. 2014 Aug 28;10(8):e1004533. doi: 10.1371/journal.pgen.1004533 (PMC4148191; doi:10.1371/journal.pgen.1004533)
Supplement: Text S1 — Supplemental materials and methods. Complete lists of alleles and transgenic lines, RT-qPCR primers, primers used to create the mutated transgenes for lin-41 and the accession numbers for proteins used in alignments in this study are provided. (PDF) [file pgen.1004533.s011.pdf]

SUPPLEMENTAL MATERIALS AND METHODS

Alleles and transgenic lines used in this study:

**CGC or lab number**	**Genotype**
1284	*rrrSi199[Pvet-4::NLS:gfp:gfp::vet-4 3'UTR; unc-119(+)] (II); rrrSi198[Pvet-4::NLS:gfp:gfp::vet-4 3'UTR; unc-119(+)] (IV)*
1180	*lin-41(rrr3)/unc-29(e1072) lin-11(n1281) (I); rrrSi199 (II); rrrSi198 (IV)*
MT7897	*lin-41(n2914)/unc-29(e1072) lin-11(n1281) (I)*
OH441	*otIs45[unc-119::gfp] (V)*
1283	*lin-41(n2914) (I); otIs45 (V)*
1252	*ojIs1[tbb-2::gfp], derived from TY3558*
1282	*lin-41(rrr3) (I); ojIs1*
1285	*lin-41(rrr4) (I); rrrSi199 (II); rrrSi198 (IV)*
1414	*lin-41(n2914) (I); rrrSi308[Plin-41::lin-41(*C114S, C117S, C130S, C151S, C154S*)::lin-41 3'UTR; unc-119(+)] (II)*
1515	*lin-41(rrr3)/hT2[qIs48] (I;III); lin-29(n546)/mnC1[dpy-10(e128) unc-52(e444) nIs190] (II)*
RW10993	*unc-119(ed3) (III); itIs37[Ppie-1::mCherry:h2b::pie-1 3'UTR; unc-119(+)]; stIs10116[Phis-72::his-24:mCherry::let-858 3'UTR; unc-119(+)]; wgIs94[Ppal-1::TY1:eGFP:3xFLAG(P000006_G02)]*
1328	*lin-41(rrr3) (I); rrrSi197[Pvet-4::NLS:mCherry:h2b::vet-4 3'UTR; unc-119(+)] (II) ; ojIs1*
1320	*lin-41(rrr3) (I); [Plin-41::flag:gfp:lin-41::lin-41 3'UTR; unc-119(+)] (II)*

1334	*lin-41(rrr3) (I); rrrSi304[Plin-41::flag:gfp:ΔNHL::lin-41 3'UTR; unc-119(+)] (II)*
1344	*lin-41(rrr3) (I); rrrSi309[Plin-41::flag:gfp:Y941A::lin-41 3'UTR; unc-119(+)] (II)*
1412	*lin-41(rrr3)/+ (I); rrrSi298[Phsp-16.41::flag:gfp:lin-41::lin-41 3'UTR; unc-119(+)] (II)*
1468	*lin-41(ma104) (I); rrrSi199[Pvet-4::NLS:gfp:gfp::vet-4 3'UTR; unc-119(+)] (II)*

Real-time quantitative PCR on dissected gonads

Primers used:

act-1 FW CTATGTTCCAGCCATCCTTCTTGG

act-1 RV TGATCTTGATCTTCATGGTTGATGG

tbb-2 FW GCTCATTCTCGGTTGTACCA

tbb-2 RV TGGTGAGGGATACAAGATGG

vet-1 FW AAAGAACTGAAACTATGTTTGCTG

vet-1 RV CTCTCGTCGTGTTTTCTGATG

vet-4 FW AAGGATTTCACTGCTTGCTC

vet-4 RV CGTCGTTTTCGATTTCTCCG

vet-6 FW GTGCGAGACAAGAATGTAATCC

vet-6 RV TTCTTGAACTCTTGGAACACAG

pes-10 FW	GCGATGATTTCATGATTTCCTG
pes-10 RV	AATTTCGTAGTCAATCTGCTCC
hlh-1 FW	ACGATTATGTGACTTCCTCTC
hlh-1 RV	GATGATCTCTATCGTCGTCC
unc-120 FW	GGGTATTATGAAGAAGGCATTCG
unc-120 RV	TGCATATGTGTAGACATGACCA
end-1 FW	GGGCAATACTTTGTTCAATCG
end-1 RV	GGATACTGTTGTGAGTAGCA
end-3 FW	GCCTATTAATGACCTCCAGC
end-3 RV	CCCGTCAATTGGTATCTCTG
elt-2 FW	AGTAAACGGAGGAATGATGTG
elt-2 RV	CTGCTCTGAAGGTATTTCCA
pha-4 FW	CCAGAATTTCCTGAACAACAC
pha-4 RV	GTTGGTGGAGCTGTAAAGAG
tbx-2 FW	AAGTGGAAGACGGATATTCC
tbx-2 RV	TTGTAACGGTGTTCATCAGC
mab-5 FW	TTCATCAAATCCATTCGCCT
mab-5 RV	CATGGAAATACTGGTTGCGA
ceh-13 FW	CAGCATAACACATACAAGTGG

ceh-13 RV GAAGTTGGTTCGATTTGTTCC

php-3 FW TTATCAAGGACACAAGCGGA

php-3 RV ATTGACATACCACTGCTCGT

hGAPDH FW GGAGTCAACGGATTTGGTC

hGAPDH RV AAACCATGTAGTTGAGGTC

Mutating LIN-41

The following primer pairs were used to introduce the point mutations:

Fw_C130S GAGTGCTGCCGTTCGTTTCAGCGCACAAGTGGGGTTTC

Rv_C130S GAAACCCCACTTGTGCGCTGAAACGAACGGCAGCACTC

Fw_C114S_C117S GCAAGACTCCTTTCGGTCCTCAGTCTCCTCCAAGAGCTCGAC

Rv_C114S_C117S GTCGAGCTCTTGGAGGAGACTGAGGACCGAAAGGAGTCTTGC

Fw_C151S_C154S GAATTTTAGCTTCCAAGCTGTCAGGTGCCGTATCGACAGCC

Rv_C151S_C154S GGCTGTCGATACGGCACCTGACAGCTTGGAAGCTAAAATTC

Fw_Y941A GTTGGCTACTTCAACGCTCCATGGGGAGTTGC

Rv_Y941A GCACATCCCCATGGAGCGTTGAAGTAGCCAAC

The following primer pair was used to create the NHL deletion mutant:

Fw_ΔNHL GATCCGGTGACGGAGAATAGACACTTTCTTCTTGC

Rv_ΔNHL GCAAGAAGAAAGTGTCTATTCTCCGTCACCGGATC

Accession numbers of proteins used in alignments

Q3B891 (UniProt). Protein: BRCA1. Species: *Homo sapiens*.

Q99496 (UniProt). Protein: RING2. Species: *Homo sapiens*.

Q13489 (UniProt). Protein: BIRC3. Species: *Homo sapiens*.

Q8WY64 (UniProt). Protein: MYLIP. Species: *Homo sapiens*.

P22681 (UniProt). Protein: CBL. Species: *Homo sapiens*.

E3MFJ2 (UniProt). Protein: LIN-41. Species: *Caenorhabditis remanei*.

G0MLY8 (UniProt). Protein: LIN-41. Species: *Caenorhabditis brenneri*.

Q9U489 (UniProt). Protein: LIN-41. Species: *Caenorhabditis elegans*.

Q2Q1W2 (UniProt). Protein: TRIM71. Species: *Homo sapiens*.

E1BJS7 (UniProt). Protein: TRIM71. Species: *Bos taurus*.

D3ZVM4 (UniProt). Protein: TRIM71. Species: *Rattus norvegicus*.

Q1PRL4 (UniProt). Protein: TRIM71. Species: *Gallus gallus*.

F6QEU4 (UniProt). Protein: TRIM71. Species: *Xenopus tropicalis*.

E7FAM5 (UniProt). Protein: TRIM71. Species: *Danio rerio*.

Q9V4M2 (UniProt). Protein: WECH. Species: *Drosophila melanogaster*.

1Q7F (PDB ID). Protein: Brat. Species: *Drosophila melanogaster*.
